# Causality between depression and ankylosing spondylitis in a European population: Results from a Mendelian randomization analysis

**DOI:** 10.1097/MD.0000000000035127

**Published:** 2023-09-22

**Authors:** Naidan Zhang, Chunjiao Song, Chaixia Ji, Baibing Xie, Yao Shu, Chengliang Yuan

**Affiliations:** a Department of Clinical Laboratory, Peoples Hospital of Deyang City, Deyang, China; b Department of Medical Technology, Chengdu Medical College, Chengdu, China.

**Keywords:** ankylosing spondylitis, depression, inverse variance weighted, mendelian randomization analysis, mendelian randomization Egger

## Abstract

The aim of this study was to explore the application of Mendelian randomization (MR) Egger and inverse variance weighted (IVW) in a causal effect on depression and ankylosing spondylitis (AS). Instrumental variables (IVs) were determined using genome-wide association studies. The 2-sample MR analysis was conducted by MR Egger to test the causal effect between depression and AS. The pleiotropy of potential instrumental variables was evaluated. The results of MR Egger and IVW were further compared. A total of 3 single nucleotide polymorphisms as the construct IVs were included. IVW results showed a significant causal effect between depression and AS (*P* < .001). Depression could promote the risk of AS (odds ratio = 1.060, 95% confidence interval: 1.026–1.094). However, the MR Egger showed no causal effect (*P* = .311). Heterogeneity statistics suggested that no heterogeneity was existed (*P* > .05). It was also suggested that there was no horizontal pleiotropy in IVs (MR Egger intercept: −0.0004, *P* = .471). Reverse MR analysis suggested that there was no causal effect between AS and depression (*P* > .05). Gene expression quantitative trait locus (QTLs) suggested that rs2517601 and RNF39 were positively correlated (beta = 1.066, *P* < .001). Depression may be one of the causes of AS by MR analysis in a European population. We can estimate the causal effect based on IVW when horizontal pleiotropy is very tiny.

## 1. Introduction

Depression is a kind of emotional reaction, which has a potential impact on human’s daily work and life.^[1]^ Ankylosing spondylitis (AS) is a chronic inflammatory disease that affects the spine, sacroiliac joints and other joints.^[2]^ At present, the etiology of AS is not clear. Factors such as genetic, infectious, environmental and immune were related to this disease.^[3–5]^ People with autoimmune diseases were more likely to develop depression than healthy controls. This association could not be fully explained by the impact of the disease on the individual.^[6]^ Meta-analysis suggested that about one-third of AS patients had depressive symptoms. The relative risk and score of depression in AS were significantly higher than healthy controls.^[7]^ However, the heterogeneity was high (*I*^2^ = 98.8%, *P* < .001). Whether depression had a causal effect on AS or not was still uncertain.

Mendelian randomization (MR) was a method that used genetic variation as instrumental variables (IVs) to infer the causal effect between exposure factors and the outcome.^[8]^ This method could effectively avoid bias caused by reverse causal association and potential confounding factors. With the increase of genome-wide association studies (GWAS), MR analysis had been widely used in epidemiological studies.^[9]^ Inverse variance weighted (IVW), as a traditional MR method, should satisfy 3 core assumptions. When the horizontal pleiotropy was existed, the estimation of causal effect was biased.^[10]^ MR Egger was a modified MR based on summary data. Different from IVW, MR Egger only needed to satisfy the instrument strength independent of direct effect and no measurement error. At the same time, this method could not only detect pleiotropy but also correct the bias.^[11]^ We used the 2 methods to explore the causal effect between depression and AS, and explained the relationship between the 2 methods.

## 2. Materials and methods

### 2.1. Sample and study flow chart

**Figure 1.**
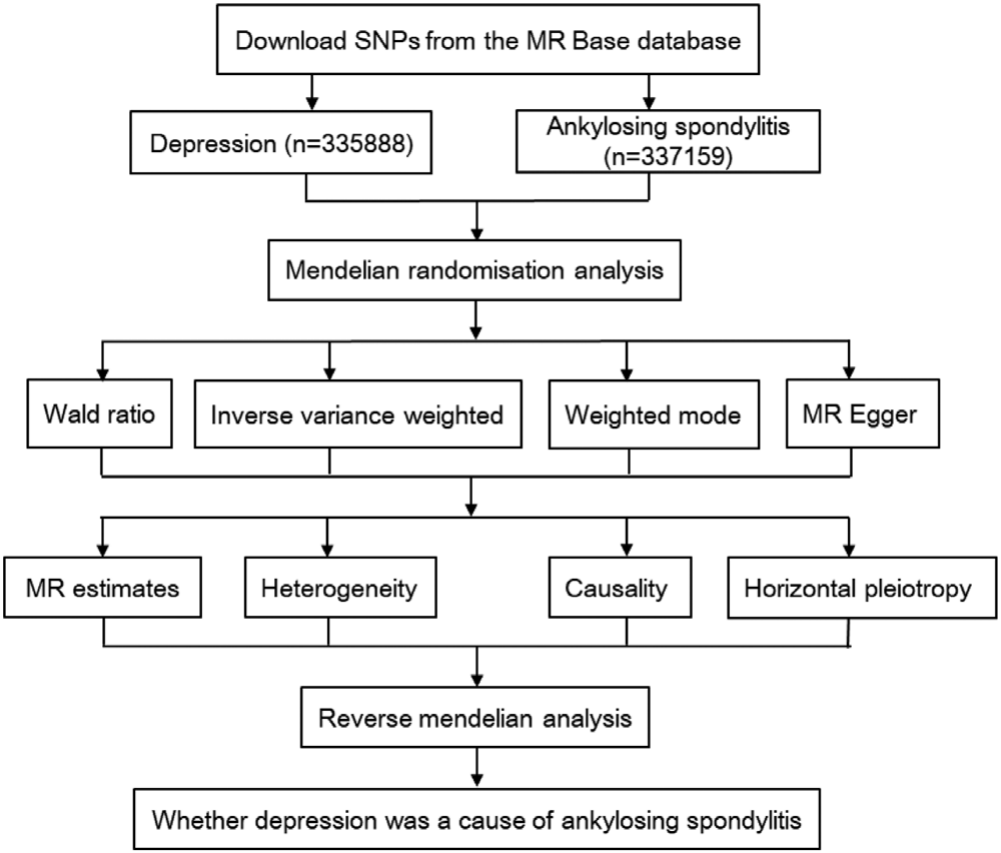
Study flowchart. MR = Mendelian randomization, SNPs = single-nucleotide polymorphisms.

We retrieved the MR Base database (http://app.mrbase.org) and performed a 2-sample MR study. In this study, R (version 4.0.3) and “TwoSampleMR” package (version 0.5.5) were used, with a test level of α = 0.05. In terms of exposure factor, we selected a GWAS of depression from Europe (n = 335888, Neale Lab Consortium). All patients had seen a psychiatrist for depression. We first assessed whether single-nucleotide polymorphisms (SNPs) were associated with depression and selected SNPs with statistical differences as IVs. At the same time, the relationship between SNPs and AS was calculated. Secondly, we combined variables to establish a 2-sample MR analysis based on “depression-SNPs-AS.” Heterogeneity and horizontal pleiotropy were evaluated. Finally, the sensitivity and bias were calculated. We also performed reverse MR to further clarify the causal effect, as shown in Figure 1.^[12]^

### 2.2. Causal effect analysis

We mainly compared the differences between “MR Egger” and “IVW” to evaluate whether there was a potential horizontal pleiotropy in MR analysis. When directionality *P* > .05, there was no horizontal pleiotropy. And the result of MR was valid. In the IVW algorithm, horizontal pleiotropy was also calculated by the intercept of the MR regression. When the intercept was close to 0, it could be ignored.

### 2.3. Heterogeneity

In order to fully evaluate the heterogeneity of SNPs, Wald ratio and forest plot were performed to get the causal effect of each SNP. Method comparison plot was used to evaluate the consistency of different methods. The funnel plot was used to evaluate the bias. The value of beta was used for odds ratio (OR), index of inconsistency (*I*^2^) and *Q* statistic. *P* < .05 was considered statistically significant.

### 2.4. Sensitivity and weak instruments

In terms of sensitivity analysis, we used leave-one-out method to eliminate SNPs one by one and calculated the combined effect of the remaining SNPs, respectively. If there was a big difference between the MR result after removing a SNP and the total result, it was indicated that the MR result was sensitive, and the hypothesis of MR was not valid. The *F* statistic was calculated to evaluate whether a single SNP would have an impact such as instrumental variable deviation. When *F* statistic < 10, it was defined as a weak bias and would affected causal effect in this study.

### 2.5. Reverse MR analysis

In order to understand whether depression and AS were mutually causal, we also conducted reverse MR analysis. We used the same 2 sets of data. The only difference was that we swapped the exposure factor and outcome. *P* < .05 was considered statistically significant.

## 3. Results

### 3.1. Characteristics of samples and IVs

This study included a GWAS of depression from Europe in 2017. The sample size was 335,888, including 38,008 patients and 297,880 controls. We analyzed 10,894,596 SNPs associated with depression. The criteria were as follows: the difference of SNPs reached genome-wide significant, namely *P* < 5 × 10^−8^; the linkage imbalance were removed with an exclusion standard of *r^2^* > 0.001 and clumping distance (kb) ≥ 10,000. The data of AS was obtained from Europe in 2017. The sample size was 337,159, including 968 patients and 336,191 controls. A total of 10,894,596 SNPs associated with AS were analyzed. All samples were none of cancer, nonspecific low back pain, lumbar disc herniation, rheumatoid arthritis, and gouty arthritis. Through 2-sample MR analysis, 3 independent SNPs were selected as IVs. The correlation between rs2517601 and AS was statistically positive (*P* = .002) (Table S1, Supplemental Digital Content, http://links.lww.com/MD/K33).

### 3.2. Result of different MR algorithms

**Figure 2.**
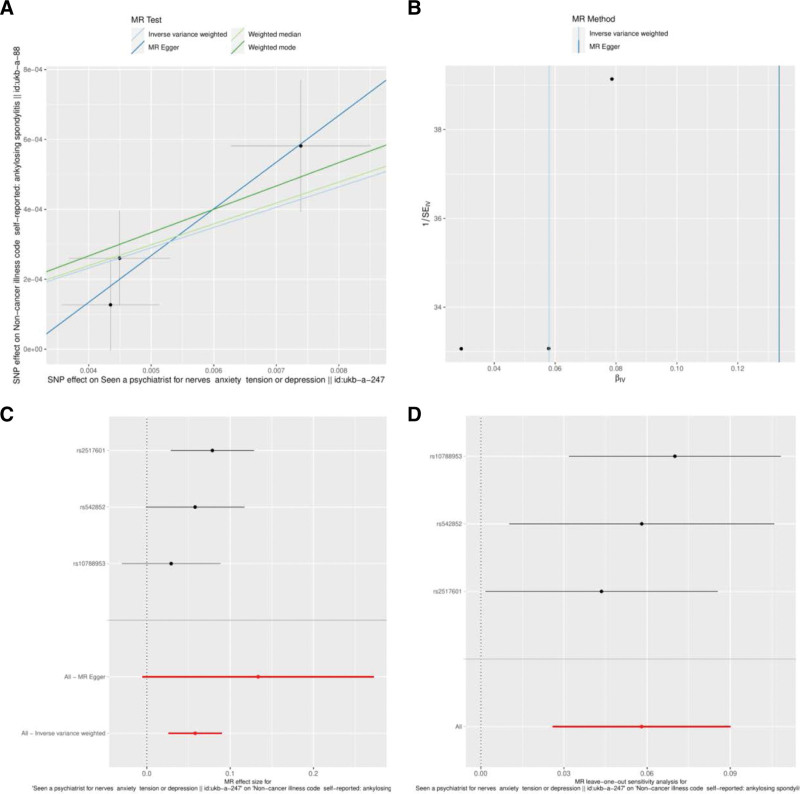
Result evaluation of MR analysis. (A) Scatter map for slopes of 4 MR algorithms; (B) funnel plot; (C) forest plot; and (D) leave-one-out. MR = Mendelian randomization, SNPs = single-nucleotide polymorphisms.

In this study, 4 algorithms of MR were used to estimate the causal effect between depression and AS. According to the scatter map, SNPs had the same influence on depression and AS. The slopes suggested that outcomes of IVW and weighted median were the closest, as shown in Figure 2A. The results of IVW (*P* < .001) and weighted median (*P* = .009) supported the causal effect. The IVW (OR = 1.060, 95% confidence interval [CI]: 1.026–1.094) suggested that the risk of AS increased by 6.0% for every 1 SD increased in depression. Weighted median (OR = 1.062, 95% CI: 1.015–1.110) suggested that the risk of AS increased by 6.2% for every 1 SD increased. The MR Egger (*P* = .311) and weighted mode (*P* = .137) did not support the causal effect between depression and AS. Meanwhile, beta of 4 algorithms were all positive, suggesting that depression could lead to an increased risk of AS, as shown in Table 1.

**Table 1:** MR regression of depression and AS.

MR method	Number of SNPs	SE	Beta	OR (95% CI)	*Q* statistic	*I* ^2^	Heterogeneity	*P*
MR Egger	3	0.071	0.134	1.143 (0.995–1.314)	0.364	1.746	0.546	.311
Weighted median	3	0.023	0.060	1.062 (1.015–1.110)				.009*
IVW	3	0.016	0.058	1.060 (1.026–1.094)	1.563	0.279	0.458	<.001*
Weighted mode	3	0.028	0.067	1.069 (1.013–1.128)				.137

AS = ankylosing spondylitis, Beta = effect sizes for each SNP, CI = confidence interval, IVW = inverse variance weighted, MR = Mendelian randomization, OR = odds ratio, SE standard errors.

* *P* < .05.

### 3.3. Pleiotropy and sensitivity

This study assessed the pleiotropy of MR, as shown in Figure 2B and C. Funnel plot and forest plot suggested that 3 SNPs in IVW were basic symmetric distribution (beta = 0.058). However, they were all on the left side in MR Egger regression (beta = 0.134). The results meant pleiotropy was existed under the MR Egger regression. According to MR Egger regression, the intercept was −4 × 10^−5^, *P* = .471, indicating that the causal effect of IVW was not affected by horizontal polymorphism in this study, as shown in Table 2.

**Table 2:** Pleiotropy and sensitivity of MR Egger.

MR method	Intercept	SE	Variance explained in exposure	*P*
MR Egger	−0.000004	0.00037	0.00031	.471

MR = Mendelian randomization, SE = standard errors.

Sensitivity analysis was performed by leave-one-out, as shown in Figure 2D and Table S2, Supplemental Digital Content, http://links.lww.com/MD/K34. The results suggested that all SNPs were greater than 0, indicating that the estimated results of MR by IVW in this study were reliable. In order to further verify the reliability of MR, we screened the weak instruments. The results indicated that the *F* statistics of all SNPs were greater than 10, which meant the IVW result was not affected by weak instruments, as shown in Table 3. We also analyzed SNPs and gene expression quantitative trait locus in the MR Base database. The results indicated that rs2517601 was positively correlated with *RNF39* (beta = 1.066, *P* < .001), as shown in Table S3, Supplemental Digital Content, http://links.lww.com/MD/K35.

**Table 3:** Screening weak instruments in IVW.

SNPs	EAF	Beta	SE	*R* ^2^	*F*	QTLs
rs10788953	0.4257	−4.35 × 10^−3^	7.79 × 10^−4^	9.26 × 10^−5^	31.1013	–
rs2517601	0.1387	−7.39 × 10^−3^	1.12 × 10^−3^	1.30 × 10^−4^	43.5725	*RNF39*
rs542852	0.6368	−4.49 × 10^−3^	8.06 × 10^−4^	9.26 × 10^−5^	31.1042	–

Beta = effect sizes for each SNP, EAF = effect allele frequency, IVW = inverse variance weighted, QTLs = quantitative trait locus, SE = standard errors, SNPs = single-nucleotide polymorphisms.

### 3.4. Results of reverse MR analysis

We used the above 4 algorithms to estimate whether there was a reverse causal effect between AS and depression. The results showed that the *P* > .05, which meant AS was not a risk factor for depression, as shown in Table 4.

**Table 4:** Reverse MR regression of AS and depression.

MR method	Number of SNPs	SE	Beta	*P*
MR Egger	7	0.115	−0.101	.419
Weighted median	7	0.010	−0.016	.872
IVW	7	0.097	−0.006	.954
Weighted mode	7	0.104	−0.017	.878

AS = ankylosing spondylitis, Beta = effect sizes for each SNP, IVW = inverse variance weighted, MR = Mendelian randomization, SE = standard errors, SNPs = single-nucleotide polymorphisms.

## 4. Discussion

Nowadays, a large number of genes have been discovered.^[13,14]^ Early MR studies tended to use a single genetic variation and focused on the risk factors with disease in a single population.^[15]^ Since many genetic variants were thought to have a pleiotropy effect, we should carefully select IVs to exclude horizontal pleiotropy when we obtained positive results by MR methods.^[16]^ IVW and MR Egger were the most common methods in MR. IVW was characterized by ignoring the existence of intercept in regression. If horizontal pleiotropy existed, the results were biased.^[17]^ The difference between MR Egger and IVW was the intercept of MR Egger. When the intercept was close to 0, it was believed that the IVW was not affected by horizontal pleiotropy. Therefore, the calculation of IVW and MR Egger was of great significance for evaluating the causal effect between exposure factors and outcome.^[18]^ For example, non-steroidal anti-inflammatory drugs (NSAIDs) were the first line of treatment for AS, which could effectively reduce pain and inflammation.^[19]^ A multicenter prospective study had also shown that combination use of NSAIDs and antidepressant improved the efficacy of antidepressant therapy.^[20]^ Did the use of NSAIDs influence the conclusion of this study? MR analysis was based on gene variation. It allowed the exposure factor of gene variation to be used as instrumental variables. And the outcome factor was predicted. Therefore, Through MR analysis, the influence of bias on causality was effectively avoided.^[21]^ In this study, the exposure factor was depression and the outcome factor was AS. NSAIDs were not the exposure factor or outcome factor. Therefore, the use of NSAIDs did not affect the conclusion.

In the actual clinical researches, we often encountered the phenomenon that the results of IVW or MR Egger were inconsistent. How do we evaluate the prediction when the result of IVW was positive and the result of MR Egger was negative? Studies have pointed out that although the MR-Egger regression has a smaller bias, its limitation is its lower statistical power in the estimation of causality.^[22]^ MR Egger regression should be viewed as a sensitivity tool to check whether instrumental variable assumptions were violated, not as a substitute for IVW.^[23]^ Although IVW was less robust, IVW estimates were more accurate. Therefore, when the results of IVW and MR Egger were inconsistent, the following 2 points should be considered as the basis for the main results and positive judgment: the results by various MR algorithms were significant, among which IVW was statistically significant, the direction of beta was consistent.^[24]^

Based on the statistical results of GWAS, SNPs closely related to depression and AS were extracted as IVs. Results of IVW showed that there was a causal effect between depression and AS. However, the MR Egger regression considered the influence of pleiotropy, and an inconsistent estimated result was given. This is consistent with the results of previous studies on MR.^[25]^ Subsequently, our detection of heterogeneity and weak IVs supported the prediction results of IVW. Why MR Egger gave inconsistent results, we speculated that it might be related to the small number of SNPs screened in this study.

For the 3 SNPs of significance in this study, analysis of SNPs and gene expression quantitative trait locus were conducted. The result indicated that rs2517601 was positively correlated with E3 ubiquitin ligase *RNF39. RNF39* was located in the MHC-I region.^[26]^ Studies have suggested that a large number of immune-related genes were in MHC-I region, such as rheumatoid arthritis and systemic lupus erythematosus.^[27,28]^ *RNF39* mediated polyubiquitination of k48 junctions and promoted the proteasome degradation of DEAD-box RNA helicase DDX3, an important scaffold of mitochondrial antiviral signaling protein/tumor necrosis factor receptor-associated factor 3 complex formation.^[29]^ New evidence suggested that *RNF39* was a potential immunomodulator. Genetic variation and hypomethylation of *RNF39* were associated with a variety of autoimmune diseases, such as Bechet’s disease, systemic lupus erythematosus and allergic rhinitis.^[30–32]^ In addition, studies on posttraumatic stress disorder suggested that hypomethylation of *RNF39* during exposure to combat trauma was associated with an increased susceptibility to posttraumatic stress disorder.^[33]^ Another study suggested that *RNF39* could lead to 22q11.2 deletion syndrome-related schizophrenia spectrum disorder by activating synaptic plasticity.^[34]^ Based on above studies, we believed that abnormal methylation of *RNF39* was associated with the occurrence of psychiatric disorders. In the follow-up study, we will conduct further researches on *RNF39* in AS patients with depression to provide more clinical evidence.

The significance of this study is to explore the causal relationship between depression and AS. Chronic pain and depression are important aspects of arthritis that are often overlooked in clinical evaluation and treatment. Through this study, we hope that attention should be paid to the evaluation of the mental aspects in arthritis patients, which may have a positive effect on improving the symptoms of arthritis. At the same time, we also note the limitations of this study. First, the research population is European population, so the results need to be verified and evaluated in multiple regions. Second, the outcome factor of AS should be refined by disease activity. This will be of guiding value to clinicians.

In conclusion, depression may be one of the causes of AS by MR analysis in a European population. We can estimate the causal effect based on IVW when horizontal pleiotropy is very tiny.

## Acknowledgements

We expressed our sincere thanks to all the patients who participated in this study.

## Author contributions

**Conceptualization:** Chengliang Yuan.

**Formal analysis:** Naidan Zhang, Chaixia Ji.

**Funding acquisition:** Naidan Zhang.

**Investigation:** Naidan Zhang, Chunjiao Song, Baibing Xie.

**Methodology:** Chunjiao Song, Chengliang Yuan.

**Project administration:** Naidan Zhang.

**Resources:** Yao Shu.

**Software:** Naidan Zhang.

**Supervision:** Chunjiao Song, Chaixia Ji.

**Writing – original draft:** Naidan Zhang.

**Writing – review & editing:** Naidan Zhang, Chengliang Yuan.

## Supplementary Material






