# Supplementary material for: Causality between depression and ankylosing spondylitis in a European population: Results from a Mendelian randomization analysis
Source: Medicine (Baltimore). 2023 Sep 22;102(38):e35127. doi: 10.1097/MD.0000000000035127 (PMC10519535; doi:10.1097/MD.0000000000035127)
Supplement: Supplementary file 1 [file medi-102-e35127-s001.docx]

**Table S1.** MR regression of single SNP.

| **Id.exposure** | **Id.outcome** | **Sample size** | **SNP** | **Beta** | **SE** | ***P*** |
| --- | --- | --- | --- | --- | --- | --- |
| ukb-a-247 | ukb-a-88 | 337159 | rs10788953 | .029 | .030 | .334 |
| ukb-a-247 | ukb-a-88 | 337159 | rs2517601 | .078 | .025 | .002* |
| ukb-a-247 | ukb-a-88 | 337159 | rs542852 | .057 | .030 | .055 |
| ukb-a-247 | ukb-a-88 | 337159 | IVW | .058 | .016 | < .001** |
| ukb-a-247 | ukb-a-88 | 337159 | MR Egger | .133 | .071 | .311 |

MR = Mendelian randomization, SNPs = single-nucleotide polymorphisms, IVW = inverse variance weighted, Beta = effect sizes for each SNP, SE = standard errors. **P* < .05, ***P* < .001.
