# Supplementary material for: Causality between depression and ankylosing spondylitis in a European population: Results from a Mendelian randomization analysis
Source: Medicine (Baltimore). 2023 Sep 22;102(38):e35127. doi: 10.1097/MD.0000000000035127 (PMC10519535; doi:10.1097/MD.0000000000035127)
Supplement: Supplementary file 2 [file medi-102-e35127-s002.docx]

**Table S2.** Results of sensitivity analysis.

| **SNPs** | **Beta** | **SE** | ***P*** |
| --- | --- | --- | --- |
| rs10788953 | .070 | .020 | < .001** |
| rs2517601 | .044 | .021 | .042* |
| rs542852 | .058 | .024 | .017* |
| All SNPs | .058 | .016 | < .001** |

SNPs = single-nucleotide polymorphisms, Beta = effect sizes for each SNP, SE = standard errors. **P* < .05, ***P* < .001.
