# Supplementary material for: Causality between depression and ankylosing spondylitis in a European population: Results from a Mendelian randomization analysis
Source: Medicine (Baltimore). 2023 Sep 22;102(38):e35127. doi: 10.1097/MD.0000000000035127 (PMC10519535; doi:10.1097/MD.0000000000035127)
Supplement: Supplementary file 3 [file medi-102-e35127-s003.docx]

**Table S3.** SNPs and QTLs in the MR Base database.

| **SNP** | **Gene name** | **Beta** | **SE** | ***P*** |
| --- | --- | --- | --- | --- |
| rs10788953 | - | - | - | - |
| rs2517601 | *RNF39* | 1.06593 | .1847 | < .001** |
| rs542852 | - | - | - | - |

SNPs = single-nucleotide polymorphisms, QTLs = quantitative trait locus, MR = Mendelian randomization, Beta = effect sizes for each SNP, SE = standard errors. ***P* < .001.
